# Core Outcome Set-STAndards for Development: The COS-STAD recommendations

**DOI:** 10.1371/journal.pmed.1002447

**Published:** 2017-11-16

**Authors:** Jamie J. Kirkham, Katherine Davis, Douglas G. Altman, Jane M. Blazeby, Mike Clarke, Sean Tunis, Paula R. Williamson

**Affiliations:** 1 MRC North West Hub for Trials Methodology Research, Department of Biostatistics, University of Liverpool, Liverpool, United Kingdom; 2 Centre for Statistics in Medicine, Nuffield Department of Orthopaedics, Rheumatology & Musculoskeletal Sciences, University of Oxford, Oxford, United Kingdom; 3 MRC ConDuCT II Hub for Trials Methodology Research, School of Social & Community Medicine, University of Bristol, Bristol, United Kingdom; 4 Northern Ireland Hub for Trials Methodology Research, Centre for Public Health, Queen's University Belfast, Belfast, United Kingdom; 5 Center for Medical Technology Policy, Baltimore, Maryland, United States of America

## Abstract

**Background:**

The use of core outcome sets (COS) ensures that researchers measure and report those outcomes that are most likely to be relevant to users of their research. Several hundred COS projects have been systematically identified to date, but there has been no formal quality assessment of these studies. The Core Outcome Set-STAndards for Development (COS-STAD) project aimed to identify minimum standards for the design of a COS study agreed upon by an international group, while other specific guidance exists for the final reporting of COS development studies (Core Outcome Set-STAndards for Reporting [COS-STAR]).

**Methods and findings:**

An international group of experienced COS developers, methodologists, journal editors, potential users of COS (clinical trialists, systematic reviewers, and clinical guideline developers), and patient representatives produced the COS-STAD recommendations to help improve the quality of COS development and support the assessment of whether a COS had been developed using a reasonable approach. An open survey of experts generated an initial list of items, which was refined by a 2-round Delphi survey involving nearly 250 participants representing key stakeholder groups. Participants assigned importance ratings for each item using a 1–9 scale. Consensus that an item should be included in the set of minimum standards was defined as at least 70% of the voting participants from each stakeholder group providing a score between 7 and 9. The Delphi survey was followed by a consensus discussion with the study management group representing multiple stakeholder groups. COS-STAD contains 11 minimum standards that are the minimum design recommendations for all COS development projects. The recommendations focus on 3 key domains: the scope, the stakeholders, and the consensus process.

**Conclusions:**

The COS-STAD project has established 11 minimum standards to be followed by COS developers when planning their projects and by users when deciding whether a COS has been developed using reasonable methods.

## Abstract 2

Paula Williamson and colleagues report on the core outcome set standards for development that researchers should use for improving reporting of their research.

## Introduction

The selection of appropriate outcomes in clinical trials and systematic reviews needs greater attention from the scientific community, if study findings are to be useful, reliable, and relevant to patients, healthcare professionals, and others making decisions regarding healthcare provision. Core outcome sets (COS) are an agreed standard set of outcomes that should be measured and reported, as a minimum, in all clinical trials in specific areas of health or healthcare [1]. The main rationales for COS are the need to improve comparability across similar trials, reduce selective outcome reporting, and increase the relevance of results from trials and systematic reviews.

The Core Outcome Measures in Effectiveness Trials (COMET) Initiative has systematically identified several hundred published COS [2–4] and registered more than 150 ongoing or planned studies in a free, publicly available, searchable database [5]. Issues to consider in COS development have been described [1], but the key elements for good-quality COS have not previously been identified through any systematic process involving all relevant stakeholders. The COMET systematic reviews [2–4] have identified variability in several aspects of COS development, including the scope, the stakeholders, and the consensus process.

Defining the quality of a COS is not straightforward. In principle, a ‘good’ COS is one that is implemented and leads to improved outcomes for patients, but this impact will be far downstream of the development process and cannot be assessed from information on the project that developed the COS [6]. In this article, we present the results of a research project to identify a set of minimum standards for COS development. These standards will help COS developers to improve their methodological approach and help users to assess whether a particular COS has been well developed.

### Scope of COS-STAD recommendations

No gold standard method for the development of a COS currently exists, although empirical evidence is starting to appear [6]. Given the number of new COS projects being registered in the COMET database, and the growing experience of COS development, it was timely to assess whether international agreement regarding design principles could be reached. The aim of the COS-STAD project was to identify those aspects of COS development for which minimum standards can be agreed upon and applied regardless of the specific consensus method chosen. The COS-STAD recommendations relate to the development process and assume that the need for a COS has already been established [6]. COS-STAD recommendations are relevant to all COS, regardless of the area of healthcare and whether the COS was developed for effectiveness trials, systematic reviews, or routine care. In parallel with the Core Outcome Set-STAndards for Reporting (COS-STAR) reporting guideline [7], the COS-STAD recommendations were developed to address the first stage of development, namely gaining agreement on what should be measured, recognising that a COS describes what should be measured in a particular research or practice setting, with subsequent work needed to determine how each outcome should be defined or measured. The distinction between COS-STAD and COS-STAR is that COS-STAD focusses on the principles of design associated with COS development, while COS-STAR relates to the reporting of COS development studies.

### Ethical approval

Ethical approval was granted for COS-STAR under an expedited review agreement (Reference RETH000841). Since the process for identifying and approaching participants to take part in COS-STAD was the same as for COS-STAR, no separate research ethics approval was sought. Informed consent was assumed if a participant responded to the surveys.

### Development of the COS-STAD recommendations

Study group members (COS-STAR authors and consensus meeting participants) were invited via an online survey to specify which aspects they considered most important when developing a COS. Participants were provided with a copy of the COS-STAR reporting guideline items as a framework for organizing possible suggestions. The survey question asked, ‘Please list the “minimum standards” that you think are important when developing a COS’. Each respondent could provide as many items as he or she wished. Responses to the survey were reviewed independently by 2 experienced COS developers (JJK and PRW), duplicate suggestions were removed, and the remaining responses were grouped into domains. Respondents were asked to clarify suggestions that were unclear or ambiguous. An explanation of the process for converting participant suggestions into domains and a preliminary list of items are provided in S1 Text. The preliminary list of 16 items was agreed upon after discussion with the other core members of the COS-STAD management group (DGA, JMB, MC, and ST). These items covered 3 domains: scope (4 items), stakeholders (4 items), and the consensus process (8 items).

This preliminary list was included in a Delphi survey of 4 key stakeholder groups, details of which are listed in Table 1. Participants were sent a personalised email outlining the project, together with a link to the survey, a copy of the COS-STAR reporting guideline [7], and the first update of a systematic review of COS studies [3]. All Delphi survey text was reviewed by the COMET PoPPIE (Patient Participation, Involvement and Engagement) coordinator in order to confirm the readability of the language prior to launching the survey.

**Table 1:** Stakeholder groups and participants involved in the Core Outcome Set-STAndards for Development (COS-STAD) Delphi survey.

Stakeholder group	Eligibility criteria	Number invited	Number responded in round 1 (*n*)	Number responded in round 2 (*n*, % retention)
(1) COS developers	Corresponding authors of published COS projects in the COMET database, with a request to forward the invitation to any methodologist that participated	253	105	103 (98%)
(2) Journal editors	Editors in chief of journals in which COS studies have been published or that are involved with CROWN*, which is a group of journals that endorse the uptake of COS [8]	131	43	36 (84%)
(3) COS users	(a) Principal investigators of open phase III/IV trials, commercial or noncommercial and registered on ClinicalTrials.gov (~10% random sample from all ongoing registered trials listed on 16 November 2016)	857	46	41 (89%)
	(b) Coordinating editors from 53 Cochrane Review Groups	76	33	31 (94%)
	(c) Clinical guideline developers* (G-I-N members) [9]	156	14	11 (79%)
(4) Patient representatives	Patient representatives from COMET workshops and the COMET PoPPIE working group* [10]	12	12	11 (92%)
Mixed stakeholder representatives^#^	Members of the OMERACT Executive Committee* (a group that advocates the use of COS for musculoskeletal conditions) [11]	12		
Mixed stakeholder representatives^#^	Those that registered for the COMET VI conference in Amsterdam in 2016 [12]	160		

* Invited to participate in the study by a third party, due to these being member organisations.

^#^ Participants were invited to select the most relevant stakeholder of the 4-stakeholder groups when participating in the Delphi tn1.

COMET, Core Outcome Measures in Effectiveness Trials; COS, core outcome set; CROWN, Core Outcomes in Women’s Health; G-I-N, Guidelines International Network; OMERACT, Outcome Measures in Rheumatology; PoPPIE, Public and Patient Participation, Involvement and Engagement.

Delphi participants rated the importance of each candidate item on a scale from 1 (not important) to 9 (critically important). In round 1 of the Delphi study, participants could suggest new items to be included in the second round but were asked not to suggest items considered good practice for research projects in general (e.g., obtaining ethical approval). In round 2, each participant who participated in round 1 was shown the number of respondents and the distribution of scores for each item, for all stakeholder groups separately, together with their own score from round 1. The data from the 3 COS user groups were presented separately. Four additional items suggested in round 1 were included and scored in round 2; the full list of additional items suggested in round 1 but not included in round 2 is given in S2 Text with reasons for their noninclusion. Consensus was defined a priori as requiring at least 70% of the voting participants from each stakeholder group to give a score between 7 and 9. COS developers (*n* = 103), COS users (*n* = 83; systematic reviewers *n* = 31, trialists *n* = 41, and clinical guideline developers *n* = 11), medical journal editors (*n* = 36), and patient representatives (*n* = 11) participated in both rounds. The Delphi process was conducted online and managed using DelphiManager software developed by the COMET Initiative [13]. The anonymised data from both rounds of the Delphi process, itemised by stakeholder group, are available as S1 Data.

Eight items reached consensus for all stakeholder groups and were automatically included in the final set of COS-STAD recommendations. The members of the COS-STAD Management Group were provided with a summary of the results (S1 Table) and asked to independently consider the remaining 12 items that did not reach consensus amongst all stakeholder groups, and they then voted as to whether the item should be included or not in the final set of standards. After the vote, 2 items supported by all COS-STAD Management Group members were included in the final set of standards, and 6 were excluded. The decision regarding the remaining items was made through discussion, and a further item was included, while 3 were excluded. A summary of the voting and this process is available in S2 Table.

Testing of the application of the minimum standards to assess COS projects was done by 15 PhD candidates as part of a training event in March 2017, hosted at the University of Liverpool in association with the European joint doctorate programme on Methods in Research on Research [14]. Students, all of whom were independent of the COS-STAD development process, worked in small groups to assess adherence of a published COS to the minimum standards [15]. They were also asked to comment on the content, format, and usefulness of COS-STAD. Feedback from this exercise revealed that it would be beneficial if those appraising COS against the standards had access to all publications relating to the COS development process, including the protocol if available.

### The COS-STAD recommendations

The 11 minimum standards presented in Table 2 are applicable to projects in which the aim is to decide which outcomes should be included in the COS. They do not address how those outcomes should be defined or measured, because guidance for that part of the process already exists [16]. The recommendations relate to 3 aspects of the COS development process: scope, stakeholders, and the consensus process.

**Table 2:** Core Outcome Set—STAndards for Development: The COS-STAD recommendations.

Domain	Standard number	Methodology	Notes
Scope specification	1	The research or practice setting(s) in which the COS is to be applied	COS developers should consider the details of the setting (e.g., for application in research studies or for use in routine care) that will be covered by the COS.
	2	The health condition(s) covered by the COS	COS developers should consider the details of the health conditions (e.g., treatment of rheumatoid arthritis or screening for cancer) that will be covered by the COS.
	3	The population(s) covered by the COS	COS developers should consider the details of the population (e.g., patients with advanced disease or children) that will be covered by the COS.
	4	The intervention(s) covered by the COS	COS developers should consider the details of the interventions (e.g., all interventions, drug therapy, or surgical interventions) that will be covered by the COS.
Stakeholders involved	5	Those who will use the COS in research	COS developers should involve those who will do the research that will use the COS (e.g., clinical trialists or industry).
	6	Healthcare professionals with experience of patients with the condition	COS developers should involve those healthcare professionals who would be able to suggest important outcomes (e.g., clinical experts, practitioners, and investigators with particular experience in the condition).
	7	Patients with the condition or their representatives	COS developers should involve those who have experienced or who are affected by the condition (e.g., patients, family members, and carers).
Consensus process	8	The initial list of outcomes considered both healthcare professionals’ and patients’ views.	COS developers should consider the views of healthcare professionals and patients (most likely identified from literature reviews or interviews) when generating an initial list of outcomes for inclusion in the consensus process.
	9	A scoring process and consensus definition were described a priori.	Although different consensus methods may be employed in different studies, to avoid any potential biases, COS developers should describe their consensus method a priori.
	10	Criteria for including/dropping/adding outcomes were described a priori.	COS developers should also prespecify criteria for including, dropping, or adding new outcomes to avoid potential biases.
	11	Care was taken to avoid ambiguity of language used in the list of outcomes.	COS developers should consider the language used when describing outcomes in front of different stakeholder groups. An example of 1 approach taken is to include both lay and medical terms, with these previously piloted with the stakeholders.

COS, core outcome set.

#### Domain 1: Scope

The scope should be defined in terms of the research or practice settings (standard 1) in which the COS is to be applied, health conditions (standard 2), target populations (standard 3), and interventions (standard 4). No recommendation is made about whether the scope of a COS should be narrow or broad, but rather, the recommendation made is that these four components should be considered and specified. For example, COS developers need to decide whether the COS is to be developed for research, routine care, or both (standard 1). The health conditions (standard 2) may be broad, e.g., all cancers, or more specific, e.g., prostate cancer. The population covered by the COS (standard 3) might be all patients with the condition or could be a specific subset, such as either localised or advanced prostate cancer patients. Finally, COS developers need to consider and specify whether the COS will apply to all interventions for the condition of interest or just specific intervention types, e.g., surgery, drugs, or medical devices (standard 4). Defining the scope of the COS at the outset of the consensus process should reduce subsequent difficulties that might arise from ambiguity of purpose. A clear specification will also help potential users decide on the relevance of the COS to their work.

#### Domain 2: Relevant stakeholders

Three stakeholder groups have been identified as the minimum for input into the development of a COS: those who will use the COS in research (standard 5), healthcare professionals (standard 6), and patients or their representatives (standard 7). Clinical trialists are an example of those who will use a COS in a research setting. Healthcare professionals are those who have direct involvement with the care and management of patients in the area for which the COS is being developed. In addition, in order for a COS to include outcomes that are most relevant to patients and carers, the inclusion of patients and carers in the COS development process is crucial.

#### Domain 3: Transparent consensus process

Transparency in the consensus process is important in order to assess whether COS recommendations were developed in a rigorous and unbiased way. Standards relating to the consensus process address 4 aspects. Outcomes considered during the consensus process should reflect the views from all relevant stakeholders (standard 8). COS developers may therefore want to consider a combination of approaches for generating the initial list of outcomes: for example, a list of outcomes from published clinical trials may be supplemented by a review of qualitative research studies investigating patients’ opinions or information from interviews with patients. Determining the scoring system and definition of consensus in advance (standard 9) reduces the risk of bias that could occur if the criteria are changed after seeing the results. Consensus criteria that are too relaxed may result in a long list of outcomes that potential users might not consider to be a core set, whilst too stringent criteria may result in key outcomes not reaching the threshold for inclusion. In a similar vein, the criteria for including, dropping, and adding outcomes should be defined in advance (standard 10). The language used to describe each potential outcome for the core set should be unambiguous (standard 11). When considering language, adequate consideration should be given to getting this right for those involved in the consensus process as well as for potential users, which may lead to the use of both plain language descriptions and medical terms, with these pilot tested for understanding.

## Discussion

Our intention in developing the COS-STAD recommendations is to encourage researchers to achieve at least the minimum standards for COS development and to help users assess whether a COS should be adopted in practice. Those looking to appraise and use published COS will need to use their own judgement regarding the applicability of the COS (scope) for the purpose they require. The COS-STAD recommendations are minimum standards and should not restrict COS developers in relation to other aspects of the process. For example, developers should include additional stakeholder groups in the development of the COS if it is felt that they are also relevant. The minimum standards relate to the principles that should be followed in the development of a COS regardless of the consensus method used. More information on different methods for achieving consensus and current evidence related to specific aspects of a consensus process for COS development can be found in the COMET Handbook [6].

Consensus was reached on 8 of the 20 candidate items at the end of the Delphi exercise. The COS-STAD Management Group voted and discussed the results for the remaining 12 items and concluded that 2 further items should be included because the majority of stakeholder groups agreed or were very close to agreement that the item was critically important. The item related to including/dropping/adding outcomes was added for consistency with the item related to the process for scoring outcomes. Defining the consensus and scoring criteria and the criteria for including/dropping and adding outcomes in advance (standards 9 and 10, respectively) would promote transparency in the reporting of the methods used and help avoid changing the criteria after the results have been analysed.

While making publicly available the protocol for a COS development study was not agreed upon as a minimum standard, the availability of a protocol will increase the transparency of the COS development process. Good research practice in general includes developing a protocol before the start of a study and making it publicly available on a suitable platform. Two of the other items that were not included in the final set related to how the views of multiple stakeholder groups will be taken into account in the consensus definition and how the views of multiple stakeholder groups will be taken into account when deciding whether to include, drop, or add outcomes during the consensus process. While these 2 items were deemed important by some stakeholder groups in the Delphi exercise, the COS-STAD Management Group did not include these explicitly because it was felt that they could already be covered by standards 9 and 10. Finally, some participants in round 1 of the Delphi process suggested an additional item, namely, that a systematic review should be undertaken to identify outcomes to be included in the consensus process. This was included for scoring in round 2 of the Delphi but only reached consensus in half of the stakeholder groups. This item was discussed by the COS-STAD Management Group but not included because it was felt that whilst a systematic review may be desirable, it could not yet be deemed essential in the absence of evidence that other forms of review or gathering of the initial list of outcomes would not suffice.

COS-STAD focusses on the main design principles for COS development, while COS-STAR [7] is exclusive to the reporting of COS studies. While COS-STAD might be used at the beginning of a COS development project and COS-STAR at the end, synergy exists between these 2 guidance documents. For example, there would be an expectation that important design principles should be reported, and since part of the methodology was for COS-STAD participants to consider the COS-STAR reporting guideline when generating the initial list of items, we have been able to deliver a set of minimum standards that are coherent with the relevant reporting guideline.

It is clear that few existing published COS would meet all 11 standards. COS methodology and consensus methodology have developed over recent years. As an example, the involvement of patients or their representatives is an area in which improvement is needed [4]. Rather than initiating new COS studies, additional work could be undertaken to supplement existing COS—for example, engagement with patients for those COS studies that did not originally include patients could be undertaken to improve existing pieces of work and to then meet this current minimum standard.

COS-STAD might be useful for users in healthcare areas in which there are several published COS for the same condition. For example, there are at least 4 COS projects in childhood asthma, with each using a different methodology and proposing slight variations in the core outcomes [17–20].

Although the acceptance rate from the Delphi invitation may appear low (Table 1), continued participation in round 2 was high for all stakeholder groups, with no evidence of attrition bias. Informed by the Delphi results, the COS-STAD Management Group decided on the final set of minimum standards. Bringing experts together for a formal consensus meeting to discuss only a small number of items was not considered to be worthwhile, particularly considering that the expertise of the Management Group covered all stakeholder groups with the exception of patient representatives. To address this limitation, we intend to work through the COMET PoPPIE Group, to actively disseminate the minimum standards to patient organisations and encourage their feedback.

There is growing concern about the relevance of some COS to research in low- and middle-income countries, since participation in COS studies in those areas has been limited. The location of many participants involved in COS development has primarily been the United States, the United Kingdom, Canada, and other European countries [21]. In the COS-STAD project, we did invite participants from global lists, but we did not collect information on the geographical location of responders. We have no reason to think that the design principles covered by the COS-STAD recommendations would not apply to all countries.

We welcome feedback on the COS-STAD recommendations. Future work is planned to explore whether criteria can be developed to identify those COS that have been developed using high-quality methods. Readers are invited to submit comments and criticisms, especially those based on experience and research evidence, via the COMET website [22]. These will be considered for future refinement of these recommendations.

## Supporting information

S1 Data Delphi Data for round 1 and round 2 of the Core Outcome Set-STAndards for Development (COS-STAD) Delphi survey. (XLSX)

S1 Table Consensus matrix for round 1 and round 2 of the Core Outcome Set-STAndards for Development (COS-STAD) Delphi survey. (DOCX)

S2 Table Core Outcome Measures in Effectiveness Trials (COMET) management group voting and discussion on items not reaching the consensus criteria from the Delphi survey by all stakeholder groups. (DOCX)

S1 Text Initial list of items suggested in the open survey of Core Outcome Set-STAndards for Reporting (COS-STAR) authors and consensus meeting participants. (DOCX)

S2 Text List of additional items suggested by participants in round 1 of the Delphi, with reasons for excluding in round 2. (DOCX)

## Abbreviations

COMET: Core Outcome Measures in Effectiveness Trials
COS: core outcome set(s)
COS-STAD: Core Outcome Set-STAndards for Development
COS-STAR: Core Outcome Set-STAndards for Reporting
PoPPIE: Public and Patient Participation, Involvement and Engagement

## References

[1] Williamson PR, Altman DG, Blazeby JM, Clarke M, Devane D, Gargon E, et al Developing core outcome sets for clinical trials: issues to consider. Trials 2012; 13:132 doi: 10.1186/1745-6215-13-132 22867278 10.1186/1745-6215-13-132 PMC3472231

[2] Gargon E, Gurung B, Medley N, Altman DG, Blazeby JM, Clarke M, et al Choosing Important Health Outcomes for Comparative Effectiveness Research: A Systematic Review. PLoS ONE 2014; 9(6): e99111 doi: 10.1371/journal.pone.0099111 24932522 10.1371/journal.pone.0099111 PMC4059640

[3] Gorst SL, Gargon E, Clarke M, Blazeby JM, Altman DG, Williamson PR. Choosing important health outcomes for comparative effectiveness research: an updated review and user survey. PLoS ONE 2016; 11(1): e0146444 doi: 10.1371/journal.pone.0146444 26785121 10.1371/journal.pone.0146444 PMC4718543

[4] Gorst SL, Gargon E, Clarke M, Smith V, Williamson PR. Choosing important health outcomes for comparative effectiveness research: an updated review and identification of gaps. PLoS ONE 2016; 11(12):e0168403 doi: 10.1371/journal.pone.0168403 27973622 10.1371/journal.pone.0168403 PMC5156438

[5] COMET Initiative Database Search 2017. [ONLINE]. Available at: http://www.cometinitiative.org/studies/search. [Accessed 15 May 2017].

[6] Williamson PR, Altman DG, Bagley H, Barnes KL, Blazeby JM, Brookes ST, et al The COMET Handbook: version 1.0. Trials 2017; 18(Suppl 3):280 doi: 10.1186/s13063-017-1978-4 28681707 10.1186/s13063-017-1978-4 PMC5499094

[7] Kirkham JJ, Gorst S, Altman DG, Blazeby JM, Clarke M, Devane D, et al Core outcome set–standards for reporting: The COS-STAR Statement. PLoS Med 2016; 13(10):e1002148 doi: 10.1371/journal.pmed.1002148 27755541 10.1371/journal.pmed.1002148 PMC5068732

[8] CROWN Initiative 2017. [ONLINE]. Available at: http://www.crown-initiative.org/ [Accessed 15 May 2017].

[9] Guidelines International Network 2017. [ONLINE]. Available at: http://www.g-i-n.net/home [Accessed 15 May 2017].

[10] COMET Initiative: People and Patient Participation, Involvement and Engagement Working Group 2017. [ONLINE]. Available at: http://www.comet-initiative.org/assets/downloads/PoPPIE%20working%20group%20member%20biographies%20v2%2003-11-16.pdf [Accessed 15 May 2017].

[11] OMERACT Executive Committee 2017. [ONLINE]. Available at: https://www.omeract.org/executive_committee.php [Accessed 15 May 2017].

[12] COMET Initiative: Past Events 2017. [ONLINE]. Available at: http://www.comet-initiative.org/events/pastcomet/ [Accessed 15 May 2017].

[13] COMET Initiative Delphi Manager 2017. [ONLINE]. Available at: http://www.comet-initiative.org/delphimanager/ [Accessed 15 May 2017].

[14] Methods in Research on Research 2017. [ONLINE]. Available at: http://miror-ejd.eu/ [Accessed 15 May 2017].

[15] Harman NL, Bruce IA, Kirkham JJ, Tierney S, Callery P, O'Brien K, et al The importance of integration of stakeholder views in core outcome set development: otitis media with effusion in children with cleft palate. PLoS ONE 2015; 10(6): e0129514 doi: 10.1371/journal.pone.0129514 26115172 10.1371/journal.pone.0129514 PMC4483230

[16] Prinsen CAC, Vohra S, Rose MR, Boers M, Tugwell P, Clarke M, et al How to select outcome measurement instruments for outcomes included in a “Core Outcome Set”–a practical guideline. Trials 2016; 17:449 doi: 10.1186/s13063-016-1555-2 27618914 10.1186/s13063-016-1555-2 PMC5020549

[17] Smith MA, Leeder SR, Jalaludin B, Smith WT. The asthma health outcome indicators study. Australian and New Zealand Journal of Public Health 1996; 20 (1): 69–75. doi: 10.1111/j.1467-842X.1996.tb01340.x 8799071 10.1111/j.1467-842x.1996.tb01340.x

[18] Sinha I, Gallagher R, Williamson PR, Smyth RL. Development of a core outcome set for clinical trials in childhood asthma: a survey of clinicians, parents, and young people. Trials 2012; 13:103 doi: 10.1186/1745-6215-13-103 22747787 10.1186/1745-6215-13-103 PMC3433381

[19] Reddel HK, Taylor DR, Bateman ED, Boulet LP, Boushey HA, Busse WW, et al An official American Thoracic Society/European Respiratory Society statement: asthma control and exacerbations: standardizing endpoints for clinical asthma trials and clinical practice. Am J Respir Crit Care Med. 2009; 180(1):59–99. doi: 10.1164/rccm.200801-060ST 19535666 10.1164/rccm.200801-060ST

[20] Busse WW, Morgan WJ, Taggart V, Togias A. Asthma outcomes workshop: overview. Journal of Allergy & Clinical Immunology 2012; 129 (3) Suppl: S1–8. doi: 10.1016/j.jaci.2011.12.985 22386504 10.1016/j.jaci.2011.12.985 PMC4259286

[21] Williamson PR. 26th Bradford Hill Memorial Lecture: Improving health by improving trials: from outcomes to recruitment and back again [ONLINE] Available at: https://www.lshtm.ac.uk/newsevents/events/26th-bradford-hill-memorial-lecture [Accessed 30 August 2017].

[22] COMET Initiative: Contact Us 2017. [ONLINE] Available at: http://www.comet-initiative.org/contactus [Accessed 15 May 2017].

