# Supplementary material for: Core Outcome Set-STAndards for Development: The COS-STAD recommendations
Source: PLoS Med. 2017 Nov 16;14(11):e1002447. doi: 10.1371/journal.pmed.1002447 (PMC5689835; doi:10.1371/journal.pmed.1002447)
Supplement: S1 Table — (DOCX) [file pmed.1002447.s002.docx]

**COS-STAD**

CONSENSUS IN (shaded in green)

Consensus definition: Support from at least 70% of participants scoring ‘Critical’, i.e. score 7-9 [from a 1-9 scale]. Participants were excluded from the calculations (denominators) if they did not score on an item

| Minimum Standard | Round 1 | | | | | | Round 2 | | | | | |
| --- | --- | --- | --- | --- | --- | --- | --- | --- | --- | --- | --- | --- |
|  | COS developer (n=105) | Journal Editor (n=43) | COS User: systematic reviewer (n=33) | COS User: trialist (n=46) | COS User: guideline developer (n=14) | Patient representative (n=12) | COS developer (n=103) | Journal Editor (n=36) | COS User: systematic reviewer (n=31) | COS User: trialist (n=41) | COS User: guideline developer (n=11) | Patient representative (n=11) |
| SCOPE: Setting | | | | | | | | | | | | |
|  | 72% | 71% | 67% | 72% | 64% | 91% | 78% | 78% | 74% | 78% | 73% | 91% |
| SCOPE: Condition | | | | | | | | | | | | |
|  | 94% | 95% | 97% | 91% | 93% | 100% | 98% | 100% | 100% | 95% | 100% | 91% |
| SCOPE: Population | | | | | | | | | | | | |
|  | 87% | 95% | 91% | 100% | 86% | 73% | 96% | 97% | 84% | 98% | 100% | 73% |
| SCOPE: Intervention | | | | | | | | | | | | |
|  | 65% | 76% | 70% | 89% | 71% | 91% | 69% | 69% | 71% | 88% | 55% | 82% |
| RELEVANT STAKEHOLDER(S): Those who will use the COS in research (e.g. clinical trialists) | | | | | | | | | | | | |
|  | 81% | 86% | 79% | 83% | 79% | 92% | 84% | 83% | 87% | 88% | 82% | 91% |
| RELEVANT STAKEHOLDER(S): Those who will use the research that should have used the COS (e.g. systematic reviewers, guideline developers, policy makers, regulatory agencies) | | | | | | | | | | | | |
|  | 55% | 74% | 82% | 67% | 79% | 83% | 52% | 78% | 81% | 64% | 73% | 73% |
| RELEVANT STAKEHOLDER(S): Healthcare professionals with experience of patients with the condition | | | | | | | | | | | | |
|  | 92% | 74% | 82% | 65% | 93% | 92% | 96% | 86% | 87% | 73% | 100% | 100% |
| RELEVANT STAKEHOLDER(S): Patients with the condition or their representatives (e.g. patients, public, participants who have experienced the condition, family members, carers) | | | | | | | | | | | | |
|  | 94% | 56% | 82% | 67% | 86% | 100% | 94% | 61% | 84% | 73% | 100% | 100% |
| TRANSPARENT CONSENSUS PROCESS: A protocol is made publically available | | | | | | | | | | | | |
|  | 57% | 65% | 76% | 58% | 71% | 58% | 56% | 69% | 71% | 63% | 73% | 73% |
| TRANSPARENT CONSENSUS PROCESS: Prospective registration of COS study in a public registry | | | | | | | | | | | | |
|  | 51% | 58% | 66% | 67% | 29% | 67% | 51% | 56% | 55% | 63% | 55% | 73% |
| TRANSPARENT CONSENSUS PROCESS: Initial list of outcomes considered *both* healthcare professionals’ and patients’ views | | | | | | | | | | | | |
|  | 76% | 67% | 79% | 70% | 64% | 83% | 85% | 86% | 84% | 77% | 82% | 91% |
| TRANSPARENT CONSENSUS PROCESS: A scoring process and consensus definition is described a priori | | | | | | | | | | | | |
|  | 81% | 74% | 79% | 80% | 86% | 100% | 78% | 75% | 77% | 85% | 100% | 100% |
| TRANSPARENT CONSENSUS PROCESS: The consensus definition specifically addresses how the views of multiple stakeholder groups will be taken into account | | | | | | | | | | | | |
|  | 75% | 63% | 70% | 64% | 71% | 83% | 77% | 64% | 71% | 62% | 73% | 91% |
| TRANSPARENT CONSENSUS PROCESS: Criteria for including/dropping/adding outcomes are described a priori | | | | | | | | | | | | |
|  | 78% | 79% | 91% | 76% | 57% | 67% | 76% | 75% | 90% | 70% | 55% | 64% |

| Minimum Standard | Round 1 | | | | | | Round 2 | | | | | |
| --- | --- | --- | --- | --- | --- | --- | --- | --- | --- | --- | --- | --- |
|  | COS developer (n=105) | Journal Editor (n=43) | COS User: systematic reviewer (n=33) | COS User: trialist (n=46) | COS User: guideline developer (n=14) | Patient representative (n=12) | COS developer (n=103) | Journal Editor (n=36) | COS User: systematic reviewer (n=31) | COS User: trialist (n=41) | COS User: guideline developer (n=11) | Patient representative (n=11) |
| TRANSPARENT CONSENSUS PROCESS: These criteria specifically address how the views of multiple stakeholder groups will be taken into account | | | | | | | | | | | | |
|  | 68% | 60% | 65% | 57% | 64% | 58% | 71% | 56% | 67% | 56% | 73% | 91% |
| TRANSPARENT CONSENSUS PROCESS: Each stakeholder group is approached in a way that should result in that stakeholder group being fairly represented | | | | | | | | | | | | |
|  | 79% | 60% | 69% | 70% | 57% | 92% | 82% | 58% | 68% | 69% | 55% | 91% |
|  | | | | | | | | | | | | |
| ADDITIONAL MINIMUM STANDARDS SUGGESTED IN DELPHI ROUND 1 THAT WERE INCLUDED IN DELPHI ROUND 2 | | | | | | | | | | | | |
|  | | | | | | | | | | | | |
| RELEVANT STAKEHOLDER(S): The research team includes representatives from all or most stakeholder groups who would use or implement the COS (e.g. patients; funders) | | | | | | | | | | | | |
|  |  |  |  |  |  |  | 54% | 61% | 65% | 40% | 55% | 73% |
| TRANSPARENT CONSENSUS PROCESS: A systematic review was undertaken to identify current outcome reporting practices | | | | | | | | | | | | |
|  |  |  |  |  |  |  | 65% | 67% | 70% | 58% | 70% | 82% |
| TRANSPARENT CONSENSUS PROCESS: Care is taken to avoid ambiguity of language used in the list of outcomes | | | | | | | | | | | | |
|  |  |  |  |  |  |  | 76% | 83% | 73% | 85% | 73% | 82% |
| TRANSPARENT CONSENSUS PROCESS: Competing interests are acknowledged in advance and taken into account during the consensus process | | | | | | | | | | | | |
|  |  |  |  |  |  |  | 63% | 72% | 94% | 65% | 73% | 82% |
