# Supplementary material for: Core Outcome Set-STAndards for Development: The COS-STAD recommendations
Source: PLoS Med. 2017 Nov 16;14(11):e1002447. doi: 10.1371/journal.pmed.1002447 (PMC5689835; doi:10.1371/journal.pmed.1002447)
Supplement: S2 Table — (DOCX) [file pmed.1002447.s003.docx]

The results (by stakeholder group) to round 2 of the Delphi survey were presented to 5 members of the COS-STAD Management Group (DA, JB, MC, PRW, ST). The eight items that reached the consensus criteria for all stakeholder groups following the Delphi exercise “Voted in at Delphi Stage” were automatically included as minimum standards. Of the remaining 12 items, the COS-STAD Management Group (MG) independently voted whether the item should be ‘IN’ or OUT’ in light of the Delphi results. Two further items were included after the initial vote “Voted in after initial vote” because all MG voted them in while six were excluded after the initial vote as all MG members voted them out “Voted out after initial vote”. The remaining four items where some MG members voted ‘IN’ and others voted ‘OUT’ were discussed further and reasons for their initial decision documented. Following discussion, three of the remaining items were voted out while a further item was voted in. The final decision reflected the initial decision of the majority, and those that initially voted in the opposite direction had no strong feelings. Red text indicates the final minimum standards.

| **Items** | % SCORING 7 - 9 BY STAKEHOLDER GROUP (Round 2 of Delphi Survey) | | | | | |  | | | | | | | | | |
| --- | --- | --- | --- | --- | --- | --- | --- | --- | --- | --- | --- | --- | --- | --- | --- | --- |
|  | COS Developers | Journal Editors | Systematic Reviewers | Trialists | Guideline Developers | Patient Representatives | COS STAD Management Group | | | | | | | | |  |
| **SCOPE** | n=103 | n=36 | n=31 | n=41 | n=11 | n=11 | Member 1 | | Member 2 | | Member 3 | | Member 4 | | Member 5 |  |
| Setting | 78% | 78% | 74% | 78% | 73% | 91% | VOTED IN AT DELPHI STAGE | | | | | | | | |  |
| Condition | 98% | 100% | 100% | 95% | 100% | 91% | VOTED IN AT DELPHI STAGE | | | | | | | | |  |
| Population | 96% | 97% | 84% | 98% | 100% | 73% | VOTED IN AT DELPHI STAGE | | | | | | | | |  |
| Intervention | 69% | 69% | 71% | 88% | 55% | 82% | IN | IN | | IN | | IN | | IN | | VOTED IN AFTER INITIAL VOTE |
| **RELEVANT STAKEHOLDER(S)** |  |  |  |  |  |  |  |  | |  | |  | |  | |  |
| Those who will use the COS in research (e.g. clinical trialists) | 84% | 83% | 87% | 88% | 82% | 91% | VOTED IN AT DELPHI STAGE | | | | | | | | |  |
| Those who will use the research that should have used the COS (e.g. systematic reviewers, guideline developers, policy makers, regulatory agencies) | 52% | 78% | 81% | 64% | 73% | 73% | IN | OUT | | OUT | | OUT | | OUT | | VOTED OUT AT CONSENSUS DISCUSSION STAGE |
| Healthcare professionals with experience of patients with the condition | 96% | 86% | 87% | 73% | 100% | 100% | VOTED IN AT DELPHI STAGE | | | | | | | | |  |
| Patients with the condition or their representatives (e.g. patients, public, participants who have experienced the condition, family members, carers) | 94% | 61% | 84% | 73% | 100% | 100% | IN | IN | | IN | | IN | | IN | | VOTED IN AFTER INITIAL VOTE |
| **TRANSPARENT CONSENSUS PROCESS** |  |  |  |  |  |  |  |  | |  | |  | |  | |  |
| A protocol is made publically available | 56% | 69% | 71% | 63% | 73% | 73% | OUT | OUT | | OUT | | OUT | | OUT | | VOTED OUT AFTER INITIAL VOTE |
| Prospective registration of COS study in a public registry | 51% | 56% | 55% | 63% | 55% | 73% | OUT | OUT | | OUT | | OUT | | OUT | | VOTED OUT AFTER INITIAL VOTE |
| Initial list of outcomes considered *both* healthcare professionals’ and patients’ views | 85% | 86% | 84% | 77% | 82% | 91% | VOTED IN AT DELPHI STAGE | | | | | | | | |  |
| A scoring process and consensus definition is described a priori | 78% | 75% | 77% | 85% | 100% | 100% | VOTED IN AT DELPHI STAGE | | | | | | | | |  |
| The consensus definition specifically addresses how the views of multiple stakeholder groups will be taken into account | 77% | 64% | 71% | 62% | 73% | 91% | OUT | OUT | | IN | | OUT | | OUT | | VOTED OUT AT CONSENSUS DISCUSSION STAGE |
| Criteria for including/dropping/adding outcomes are described a priori | 76% | 75% | 90% | 70% | 55% | 64% | IN | OUT | | IN | | OUT | | IN | | VOTED IN AT CONSENSUS DISCUSSION STAGE |
| These criteria specifically address how the views of multiple stakeholder groups will be taken into account | 71% | 56% | 67% | 56% | 73% | 91% | OUT | OUT | | OUT | | OUT | | OUT | | VOTED OUT AFTER INITIAL VOTE |
| Each stakeholder group is approached in a way that should result in that stakeholder group being fairly represented | 82% | 58% | 68% | 69% | 55% | 91% | OUT | OUT | | OUT | | OUT | | OUT | | VOTED OUT AFTER INITIAL VOTE |
| A systematic review was undertaken to identify current outcome reporting practices | 65% | 67% | 70% | 58% | 70% | 82% | OUT | OUT | | OUT | | IN | | OUT | | VOTED OUT AT CONSENSUS DISCUSSION STAGE |
| Care is taken to avoid ambiguity of language used in the list of outcomes | 76% | 83% | 73% | 85% | 73% | 82% | VOTED IN AT DELPHI STAGE | | | | | | | | |  |
| Competing interests are acknowledged in advance and taken into account during the consensus process | 63% | 72% | 94% | 65% | 73% | 82% | OUT | OUT | | OUT | | OUT | | OUT | | VOTED OUT AFTER INITIAL VOTE |
| The research team includes representatives from all or most stakeholder groups who would use or implement the COS (e.g. patients; funders; etc) | 54% | 61% | 65% | 40% | 55% | 73% | OUT | OUT | | OUT | | OUT | | OUT | | VOTED OUT AFTER INITIAL VOTE |
