# Supplementary material for: Core Outcome Set-STAndards for Development: The COS-STAD recommendations
Source: PLoS Med. 2017 Nov 16;14(11):e1002447. doi: 10.1371/journal.pmed.1002447 (PMC5689835; doi:10.1371/journal.pmed.1002447)
Supplement: S1 Text — (DOCX) [file pmed.1002447.s004.docx]

In order to generate the initial list of items, JJK and PRW independently reviewed the verbatim text of suggested items received by the COS-STAD study team. Key phrases/themes were underlined. JJK/PRW then considered each respondent text in turn and discussed their initial findings. Any duplicate suggestions across respondents were noted. A summary of the discussions is listed under ‘JJK/PRW review’. Suggestions relating to good research practice more generally or reporting items only were not considered further. We also considered only items that covered the basic principles of COS development, specific items that related to a specific method of COS development were not considered as these would not reflect minimum standards.

It became apparent from the discussion that three key domains emerged. The unique preliminary list of items was generated by taking the specific details (if provided) from the verbatim text suggested by participants under each domain. The list was reviewed and verified by the COS-STAD management group.

| **Respondent Number** | **Item suggested (verbatim text)** | **JJK / PRW Review** | **Domain** | **Preliminary list of items** |
| --- | --- | --- | --- | --- |
| 1 | *Inclusion of relevant stakeholders in the process for agreeing on the COS (including those who will do the research that will use the COS and those who will use the research that should have used the COS).* | - Relevant stakeholders   *those that will use the COS (e.g. clinical trialists)*  *those who will use the research that should have used the COS (e.g. systematic reviewers, guideline developers)* | 1. Stakeholders | 1. Those who will use the COS in research 2. Those who will use the research that should have used the COS |
| 2 | *Determine rationale for COS (e.g. heterogeneity of outcomes currently reported in specific area); Gain ethical approval; Identify detailed scope of COS (setting/population/condition/intervention); Consideration of relevant stakeholders / stakeholder subgroups to include; Determine eligibility criteria for participants and how they will be identified and invited. Consideration and rationale for chosen consensus method. Determine whether potential to introduce new outcomes during the process; If Delphi - determine number of rounds, method of feedback, what will be fed back to who and in what format; If a consensus meeting - develop rationale for the structure/participants/facilitator. What outcomes will be discussed, how consensus defined; Consideration of attrition and impact on final COS; Consideration of limitations; Consideration of implementation.* | - Scope of COS   *Setting*  *Population*  *Condition*  *Intervention*   - Relevant stakeholders   *No specifics provided*  Remaining issues are either reporting / relate to good research practice more generally or too specific (rather than basic principles) for minimum standards | Scope  Stakeholders | 1. Setting 2. Population 3. Condition 4. Intervention   No new item suggested |
| 3 | *Involvement of healthcare professionals relevant to the scope of the COS Patient participation (i.e. patient voice is heard) A review of what is known to date is undertaken - outcomes measured in trials, patients' views Consensus process is transparent - likely helped by having a protocol COS-STAR guideline is followed* | - Relevant stakeholders   *Healthcare professionals*  *Patients*   - Identification of what was known already to avoid unnecessary duplications - Transparent consensus process - Protocol is available | Stakeholders  Consensus Process | 1. Healthcare professionals with experience of patients with the condition 2. Patients with the condition or their representatives 3. A protocol is made publically available |
| 4 | *1. Clear specification of scope: health condition(s) plus whichcver of the following are relevant: population covered, specific interventions, setting(s) 2. All important stakeholder groups involved [hard too know how to define though!] 3. how initial list of potential outcomes was derived 4. Detail of the consensus process to produce final COS (implies that there was one!)* | - Scope – duplicate suggestions (from respondent 2) - Relevant stakeholders   *No specifics provided*  Items 3 and 4 here relate to reporting standards because nothing specific is specified. | Scope  Stakeholders | No new item suggested  No new item suggested |
| 5 | *Describe the health condition(s) and population(s) covered by the COS Describe the intervention(s) covered by the COS Stakeholder groups - Patients, Practitioners, Domains: Domain Information sources- Patients ,Practitoners interviews/survey;literature survey Domain Consensus process with defined pass-rate that includes equal weighting of patients and others for Domain selection Instrument selection : Existing Instruments -literature search; Match with Domains and easy- to- understand transparent scoring ;Feasibility; Cohort 2studies of Responsiveness[preferably showing discimination in RCT ; as least showing change in cohort to correlate with global score If no existing Instrument : Match with Domains and easy- to- understand transparent scoring;Feasibility; Cohort 2studies of Responsiveness[preferably showing discimination in RCT ; as least showing change in cohort to correlate with global score Ethics and consent Conflicts of interest* | - Scope – duplicate suggestions (from respondent 2) - Relevant stakeholders   *Patients – duplicate suggestions (from respondent 3)*  *Practitioners - duplicate suggestions (from respondent 3)*   - Consensus process   *A description about the scoring and consensus definition in advance*  The remainder relates to information about measurement instruments which is beyond the scope of the minimum standards. Ethics and conflicts of interest relate to good research practice more generally. | Scope  Stakeholders  Consensus Process | No new item suggested  No new item suggested   1. A scoring process and consensus definition were described a priori |
| 6 | *reproducible methods; evidence-based development; completeness and transparency in the development processes* | - Transparent consensus process - *duplicate suggestions (from respondent 3)*   No specifics provided. | Consensus Process | No new item suggested |
| 7 | *My suggestions for minimum standards for COS development, using item number from the checklist: 1a 2a, 2b 3a, 3b, 3c 8 9a, 9b 12 13a, 13b 14 17 (From COS-STAR)* | These items are from COS-STAR and related to scope, the consensus process and stakeholder involvement which are all duplicate suggestions. The remaining items are reporting issues only. | Scope  Stakeholders  Consensus Process | No new item suggested  No new item suggested  No new item suggested |
| 8 | *Patient and clinical expert participation A form of consensus methodology used Consensus defined a priori Recognition of limitations/shortcomings of COS* | - Relevant stakeholders   *Healthcare professionals - duplicate suggestions (from respondent 3)*  *Patients- duplicate suggestions (from respondent 3)*   - Transparent consensus process - *duplicate suggestions (from respondent 5)* | Stakeholders  Consensus Process | No new item suggested  No new item suggested |
| 9 | *A: specifications and statement of reasons for specifications of the following aspects need to be provided: - the condition of interest and possibly the intervention of interest (if the COS is focussing on a specific Intervention) - the SETTING (e.g. trial, registry, clinical practice, quality assurance) - the geographical scope (e.g. international / global or regional / national focus) - and the relevant stakeholders B: development of a protocol / a priori plan before conduct of COS development (relating to the items included in the COS STAR checklist C: use of appropriate evidence synthesis methods with regard to the specifications made (see A) before / as a basis for consensus excercise D: use of appropriate / transparent / fair consensus methods including the stakeholders listed under A E: development of a dissemination strategy for the COS developed F: Monitoring of the application of the COS in the setting and Population with planned action in case it is not used as intended.* | - Scope of COS   *Setting – duplicate (from respondent 2)*  *Population / geographic– duplicate (from respondent 2)*  *Condition– duplicate (from respondent 2)*  *Intervention– duplicate (from respondent 2)*   - A protocol is available - *duplicate (from respondent 3)* - Identification of what was known already - *duplicate suggestions (from respondent 3)* - Consensus process   *Each stakeholder is represented fairly*  Dissemination and monitoring are beyond the scope of the minimum standards | Scope  Consensus Process  Consensus Process | No new item suggested  No new item suggested   1. Each stakeholder group is approached in a way that should result in that stakeholder group being fairly represented |
| 10 | *Reference to literature that might inform the process Well described method for eliciting outcomes initially - ideally using patient/public as source as well as clinicians and researchers Well described criteria for inclusion and exclusion of outcomes for prioritization Well described method for prioritisation of outcomes Well described process and criteria for dropping outcomes that are not considered core Well described method for final agreement of core outcomes Inclusion of; Stakeholders especially from my perspective patients/public/family members as appropriate Clinical trial leaders and others involved in research funding and policy (so that the core outcomes stand more of a chance of being adopted) Dissemination plan for promoting core outcomes for communities of interest* | - Relevant stakeholders   *Clinicians - duplicate suggestions (from respondent 3)*  *Patient/Public- duplicate suggestions (from respondent 3)*   - Consensus process   *Initial list of outcomes considered both healthcare professionals’ and patients’ views*  *Criteria for including, excluding and dropping outcomes*  Dissemination is beyond the scope of the minimum standards. | Stakeholders  Consensus Process | No new item suggested   1. Initial list of outcomes considered *both* healthcare professionals’ and patients’ views 2. Criteria for including/dropping/adding outcomes were described a priori |
| 11 | *- Describe the rationale for stakeholder groups involved in the COS development process, eligibility criteria for participants from each group and a description of how the individuals involved were identified - Describe the information sources used.* | - Relevant stakeholders   *No specific suggestions – rationale is also not a minimum standard.*  Describing information sources is a reporting item | Stakeholders | No new item suggested |
| 12 | *Patients participation in developing the core outcome set Appropriate sample size (study population e.g. number of patients interviewed/surveyed) Selection bias: appropriate methods to minimise selection bias (both patient recruitment and selection of outcomes) Researchers conflicts of interest and funding sources The core outcomes need to be both clinically meaningful and patient important (preferably not surrogate outcomes) Consensus process: need to be a formal consensus process* | - Relevant stakeholders   *Patients- duplicate suggestions (from respondent 3)*   - Consensus process   *No specific suggestions*    Conflicts of interest and funding source relate to good research practice more generally. Sample size was considered too difficult to generalise for all COS. | Stakeholders  Consensus Process | No new item suggested  No new item suggested |
| 13 | *I could see this question applying equally to the items included in a COS, or to the research underlying the development of a COS, so have provided suggestions relevant to each. Outcomes in a COS should be, at a minimum: 1) Of demonstrated importance to people who have experienced the condition to which the COS applies 2) Able to be assessed across the settings in which application of the COS is intended; for example, a COS intended for clinical use should not requiring access to technologies available only in research settings, and a COS intended for global use should not rely on proprietary assessment tools that would be prohibitively expensive to access in low-income settings. 3) If a surrogate marker, clearly linked by substantial evidence to condition-specific clinical outcomes (eg. CD4 T-cell count in HIV) 4) Measurable with demonstrated validity (accuracy and precision) within an individual and across the population in which the COS is intended to be used 5) Minimally invasive among available options; for example, an outcome requiring CSF sampling or tissue biopsy should not be included in a COS if blood or imaging results would provide similar information 6) Sufficiently well described to permit replication, validation, re-use or modification by other researchers Projects to develop COS should, at a minimum: 1) Proceed based on a prespecified research protocol and analysis plan to be published together with the core outcome set, with explanation of any differences between the prior plan and the published article 2) Involve investigators with specific experience in care or management of the condition to which the core outcome set applies 3) Include participants who have experienced the condition to which the COS applies 4) Receive all appropriate ethical approvals (including informed consent if appropriate) prior to initiation of data collection, or prior to enrollment of the first participant if COS development is part of a clinical trial* | - A protocol is available - *duplicate (from respondent 3)* - Relevant stakeholders   *Healthcare professionals- duplicate (from respondent 3)*  *Patients -- duplicate (from respondent 3)*  Ethics relates to good research practice more generally. | Consensus Process  Stakeholders | No new item suggested  No new item suggested |
| 14 | *Design stage 1. Systematic review of existing literature according to PRISMA guidelines 2. Prospective registration / publication of study protocol Development stage 1. Use of a consensus method with appropriate justification 2. Outcome domains included in consensus stages have been identified via well conducted systematic reviews of existing literature 3. Involvement of relevant stakeholder groups 4. Appropriate justification for inclusion / exclusion of relevant groups Publication stage 1. Reporting of COS according to appropriate guidelines (COS-STAR)* | - Consensus process   *Registrations of study*  *Study protocol - duplicate (from respondent 3)*   - Identification of what was known – duplicate (from respondent 3) - Relevant stakeholders   *No specific suggestions*  COS-STAR and publication related to reporting. | Consensus Process  Stakeholders | 1. Prospective registration of COS study in a public registry |
| 15 | *Define the objectives of the COS, including health condition, population, intervention(s) and settings; Define the eligibility criteria for participants from each stakeholder group; Describe the methods by which an initial list of outcomes were identified; Describe the methods by which an initial list of outcomes were initially prioritised; Describe the consensus development process including criteria used for judging that consensus was achieved and the process for development of consensus including scoring of outcomes; Describe process for obtaining informed consent from participants and clarify if ethical approval obtained; Describe any deviations from a priori decisions in the development of the COS Describe the participants, including all participant groups, involved at each stage of the COS development Describe what happened to initial list of outcomes during interim prioritisation and the inclusion and exclusion of outcomes throughout the COS development process; List the outcomes in the final core outcome set; Identify important limitations and.or biases in the COS development process; Describe implications for practice and implications for research as appropriate; State source of funding and any conflicts of interest* | - Scope – duplicate suggestions (from respondent 2) - Relevant stakeholders   *No specific suggestions*   - Identification of what was known already *-* duplicate (from respondent 3) - Consensus process   *Criteria for including/dropping/adding outcomes were described a priori - duplicate (from respondent 10)*  *A description about the consensus definition - duplicate (from respondent 5)*  Many suggestions here are reporting issues, e.g. listing the outcomes in the final core set. Ethics, funding and conflicts of interest relates to good research practice more generally. | Scope  Stakeholders  Consensus Process | No new item suggested  No new item suggested  No new item suggested |
| 16 | *That the long list has patient input in additional to that from professionals That consensus methods are transparent That decisions to remove items or include others are transparent This applies to COS for effectiveness trials (not other sorts in which patient input less important)* | - Relevant stakeholders   *Patients -- duplicate (from respondent 3)*   - Consensus process   *Criteria for including/dropping/adding outcomes were described a priori - duplicate (from respondent 10)* | Stakeholders  Consensus Process | No new item suggested  No new item suggested |
| 17 | *This objective is a little unclear to me as I thought that's what the checklist was for - a minimum set of items to report how a COS was developed? I think "quality" should likely be avoided and "risk of bias" adopted. What are the elements that put a COS at risk of bias? I think they are risk from being influenced by the people who developed them, and the stakeholders who were/weren't asked to participate. How items were shaped/added/dropped also play a key role wrt potential to bias outcomes. I'm not sure if I'm addressing the issue you were hoping to address....* | - Relevant stakeholders   *No specifics provided*   - Consensus process   *Criteria for including/dropping/adding outcomes were described a priori - duplicate (from respondent 10)* | Stakeholders  Consensus Process | No new item suggested  No new item suggested |
| 18 | The consensus definition specifically addresses how the views of multiple stakeholder groups will be taken into account. These criteria [including/dropping/adding outcomes] specifically address how the views of multiple stakeholder groups will be taken into account | \| - Consensus process   *Consensus definition takes into account views of multiple stakeholder groups taken into account*  *Criteria for including/dropping/adding outcomes address how the views of multiple stakeholder groups will be taken into account* \| \| \| --- \| --- \| \|  \|  \| | Consensus Process | 1. The consensus definition specifically addresses how the views of multiple stakeholder groups will be taken into account 2. These criteria specifically address how the views of multiple stakeholder groups will be taken into account |
