# Supplementary material for: Core Outcome Set-STAndards for Development: The COS-STAD recommendations
Source: PLoS Med. 2017 Nov 16;14(11):e1002447. doi: 10.1371/journal.pmed.1002447 (PMC5689835; doi:10.1371/journal.pmed.1002447)
Supplement: S2 Text — (DOCX) [file pmed.1002447.s005.docx]

| **Additional item suggestions** | **JJK** | **PRW** | **Final Decision** |
| --- | --- | --- | --- |
|  |  |  |  |
| State a-priori if other core outcome sets with similar scope are already available | Reporting only – See COS-STAR | Reporting only – See COS-STAR | Do not include in round 2 of Delphi |
| If other similar core outcome sets are already available, describe the overlap with these core sets and explain the rationale for developing a new one | Reporting only – See COS-STAR | Reporting only – See COS-STAR | Do not include in round 2 of Delphi |
| Description of consensus meeting process (various potential items, discuss with OMERACT reps) | Reporting only – See COS-STAR | Reporting only – See COS-STAR | Do not include in round 2 of Delphi |
| Reporting of the COS according to COS-STAR guidelines. | Reporting only – See COS-STAR | Reporting only – See COS-STAR | Do not include in round 2 of Delphi |
| The method in which stakeholder group opinion is gathered should be reported (i.e. surveys, focus groups) | Reporting only – See COS-STAR | Reporting only – See COS-STAR | Do not include in round 2 of Delphi |
| Representatives of all key stakeholder groups (inc. patients and healthcare professionals) are involved in the development of the COS process | Already included in stakeholder domain | Already included in stakeholder domain | Do not include in round 2 of Delphi |
| Guidance on appropriate measurement of core items (e.g. timing of measures, preferred measurement tools or techniques) | Beyond scope of this research | Relates to ‘how’ and not ‘what’ to measure | Do not include in round 2 of Delphi |
| Use of systematic reviews to identify current outcome reporting practices | Include in round 2 | While I agree I think this is more quality - the minimum standard is "Initial list of outcomes considered both healthcare professionals’ and patients’ views". How developers achieve this is a separate issue | Include in round 2 of Delphi |
| Use of systematic approaches to assess validity and reliability of potential core outcomes | Beyond scope of this research | Relates to ‘how’ and not ‘what’ to measure | Do not include in round 2 of Delphi |
| Systematic literature reviews should be conducted to appraise the measurement properties of selected outcome measures (e.g. patient-reported outcomes, performance based measures) – I’m not sure if this will be addressed in a next study phase? It is not uncommon for COS studies to recommend the use of measures that lack validity evidence, which is extremely counterproductive. | Beyond scope of this research | Relates to ‘how’ and not ‘what’ to measure | Do not include in round 2 of Delphi |
| Systematic reviews should be conducted to identify standardized definitions of specific outcome measures (e.g. complications, adverse events). Again, I’m not sure if this will be addressed in a next study phase. COS only partially addresses the issue of standardization (results from studies assessing a same COS will still not be comparable if specific outcome definitions are not standardized) | Beyond scope of this research | Relates to ‘how’ and not ‘what’ to measure | Do not include in round 2 of Delphi |
| The methods for developing measurement tools for the COS should be specified in advance | Beyond scope of this research | Relates to ‘how’ and not ‘what’ to measure | Do not include in round 2 of Delphi |
| Self-care definition for patients | Unclear | Unclear | Participant suggests this relates to ‘how’ outcomes should be measured. Do not include in round 2 of Delphi |
| Linguistic Validation Standard Process | Unclear | Unclear | Participant suggests this relates to ‘how’ outcomes should be measured. Do not include in round 2 of Delphi |
| Classification of primary vs secondary clinical endpoints | Not appropriate | Not the primary objective of COS development | Do not include in round 2 of Delphi |
| Patient Reported Outcome | Already covered in another item | Already covered in another item | Do not include in round 2 of Delphi |
| Clinician Reported Outcome | Already covered in another item | Already covered in another item | Do not include in round 2 of Delphi |
| The research team includes representatives from all or most stakeholder groups who would use or implement the COS, e.g. patients, funders | Include in round 2 | Include in round 2 | Include in round 2 of Delphi |
| Consideration of Objective information over Subjective or Qualitative | To do with measurement | Relates to ‘how’ and not ‘what’ to measure | Do not include in round 2 of Delphi |
| The anticipated uses to which the COS is hoped to be relevant (should be in the scope) | Already considered in scope domain | Reporting only | Do not include in round 2 of Delphi |
| Cost (or cost reduction) of change in outcome | Unclear | Beyond scope – difficult to determine during development phase | Do not include in round 2 of Delphi |
| Criteria as to who is chosen within each stakeholder group | Reporting only – See COS-STAR | Reporting only – See COS-STAR | Do not include in round 2 of Delphi |
| Criteria on how the members of each stakeholder group are approached to participate | Reporting only – See COS-STAR | Reporting only – See COS-STAR | Do not include in round 2 of Delphi |
| There should be at least n=30 participants in each stakeholder group | Too difficult to quantify / generalise for all COS | Difficult to consider | Do not include in round 2 of Delphi |
| Participants not responding in one of the Delphi rounds, should not be asked in a next round | Assumes a Delphi was done | Assumes a Delphi was done | Do not include in round 2 of Delphi |
| The Delphi results should be fed back anonymously | Assumes a Delphi was done | Assumes a Delphi was done | Do not include in round 2 of Delphi |
| Rating of outcome measures and outcome measurement instruments should be kept separate | Beyond scope of this research | Relates to ‘how’ and not ‘what’ to measure | Do not include in round 2 of Delphi |
| Patients implication in developing COS is crucial. Not sure the stakeholder group of Journal Editor is important. | Already covered by patient item | Already covered by patient item | Do not include in round 2 of Delphi |
| Should ideally start with domains then instruments in that order | Beyond scope of this research | Relates to ‘how’ and not ‘what’ to measure | Do not include in round 2 of Delphi |
| Should involve regulators and industry as key stakeholders | Already covered in ‘those who will use the COS in research’ | Already covered in ‘those who will use the COS in research’ | Do not include in round 2 of Delphi |
| The search strategy must be made transparent | Reporting only – See COS-STAR | Reporting only – See COS-STAR | Do not include in round 2 of Delphi |
| Major unexpected outcome: incidence and some information on what investigation ensued +/- results for the investigation | Reporting only – See COS-STAR (limitation) | Unclear | Do not include in round 2 of Delphi |
| Perhaps consider the timing of when the COS will be used e.g. at a specific point in the illness/recovery trajectory in relation to when certain interventions might be used | Covered by scope criteria | Covered by scope criteria | Do not include in round 2 of Delphi |
| Criteria describing how the final consensus meeting will be conducted, especially the voting process | Reporting only – See COS-STAR | Reporting only – See COS-STAR | Do not include in round 2 of Delphi |
| Process of implementing the COS should be described | Beyond scope of COS development process | Beyond scope of COS development process | Do not include in round 2 of Delphi |
| Process of updating the COS should be described | Beyond scope of COS development process | Beyond scope of COS development process | Do not include in round 2 of Delphi |
| Allegiance of researchers should always be investigated and clear | Reporting only – See COS-STAR | Might be important to include in round 2 | Include in round 2 of Delphi |
| For transparency - weighting given to different stakeholder groups | Reporting only – See COS-STAR | Reporting only – See COS-STAR | Do not include in round 2 of Delphi |
| Plan for distribution and implementation of the COS | Beyond scope of COS development process | Possibly include in round 2 | Do not include in round 2 of Delphi |
| Plan for reviewing the COS i.e. at a certain time point as per Cochrane or if there is a substantial change to the condition/ clinical management etc. | Beyond scope of COS development process | Possibly include in round 2 | Do not include in round 2 of Delphi |
| COS should be developed by experts only, and the input of different stakeholders should be well defined and can be limited for specific stakeholders, as not all stakeholders do have the same expertise or relevance | Too vague | Too vague | Do not include in round 2 of Delphi |
| Will a consensus meeting be used | Implies there will be a consensus meeting | Implies there will be a consensus meeting | Do not include in round 2 of Delphi |
| How is the consensus meeting valued ( I think this is incorporated in the protocol question but that is not so specific) | Implies there will be a consensus meeting | Reporting only – See COS-STAR | Do not include in round 2 of Delphi |
| Approach of stakeholders (open? Snowballing? anonymous/semi anonymous) | Reporting only – See COS-STAR | Reporting only – See COS-STAR | Do not include in round 2 of Delphi |
| To help patients to speak up for themselves | Covered by patient item already | Covered by patient item already | Do not include in round 2 of Delphi |
| To use patients as a resource and not as a token( in many cases it is pinpointed the importance of patients' perspective, but at the end of the day they remained neglected | Covered by patient item already | Covered by patient item already | Do not include in round 2 of Delphi |
| Existing relevant COS development studies should be reviewed and discussed | Reporting only – See COS-STAR | Reporting only – See COS-STAR | Do not include in round 2 of Delphi |
| COS development should include discussion of appropriate measuring instruments | Beyond scope of this research | Relates to ‘how’ and not ‘what’ to measure | Do not include in round 2 of Delphi |
| COS development should include determination or at least discussion of minimal clinically relevant differences | Beyond scope of this research | Relates to ‘how’ and not ‘what’ to measure | Do not include in round 2 of Delphi |
| Consensus process facilitates communication and discussion between stakeholder groups | Too vague | Too vague | Do not include in round 2 of Delphi |
| Process is international | May not apply to all COS | May not apply to all COS | Do not include in round 2 of Delphi |
| Ambiguity of language is avoided when defining the list of outcomes | Include in round 2 | Include in round 2 | Include in round 2 of Delphi |
